# Intradiscal cement leakage (ICL) increases the stress on adjacent vertebrae after kyphoplasty for osteoporotic vertebra compression fracture (OVCF): a finite-element study

**DOI:** 10.1038/s41598-023-43375-5

**Published:** 2023-09-25

**Authors:** Hai Meng, Qiujun Li, Jisheng Lin, Yong Yang, Qi Fei

**Affiliations:** 1 Department of Orthopedics, Beijing Friendship Hospital, Capital Medical University, No 95, Yong’an Road, Xicheng District, 100050 Beijing, People’s Republic of China; 2 Department of Anesthesiology, Beijing Jishuitan Hospital, Beijing, 100035 China

**Keywords:** Diseases, Medical research

## Abstract

This study aimed to explore the biomechanical effects on adjacent vertebra of thoracolumbar Osteoporotic Vertebra Compression Fracture (OVCF) after Percutaneous Kyphoplasty (PKP) with intraoperative intradiscal cement leakage (ICL) by applying a Finite-Element Analysis. We collected pre- and post-operative computer tomography (CT) images of a 71-year-old female patient with single T12 OVCF, who underwent an intraoperative cement leakage into the T12–L1 disc. Three-dimensional finite element models of thoracolumbar spine (T10–L2) were built with the support of Materialise Interactive Medical Image Control System (MIMICS) and ABAQUS software. The stress on adjacent vertebrae and endplates under the uniform compressive pressure (0.3 MPa) and during different loading moments were analyzed. The three-dimensional finite element models reveal an asymmetrical distribution of von Mises stresses on the adjacent endplate unaffected by the surgical intervention. The maximum von Mises stress on adjacent vertebral bodies increased during different loading conditions, especially for lateral bending and rotation loading conditions, whereas the maximum von Mises stress on distal non-treated vertebrae decreased on anteflexion and backward extension loading conditions. Post-operative adjacent vertebra compression fractures after PKP with intraoperative intradiscal cement leakage (ICL) may be closely related to the biomechanical changes of adjacent vertebrae of thoracolumbar OVCF, and it may increase the risk of postoperative fracture.

## Introduction

Osteoporotic vertebral compression fracture (OVCF) represents one of the most common complications of osteoporosis. The kyphotic deformities caused by OVCF are usually manifested as pulmonary dysfunction, constipation, and imbalance that severely affect the patient’s physical status, and lead to an increased risk of mortality. Approximately 1.5 million cases of osteoporotic fracture are reported annually in the USA, and between one third and a half of them are OVCF^1^, which usually occurs at thoracolumbar and middle thoracic vertebra. The economic costs of osteoporosis and fractures in the USA reached nearly $16–$22 billion for one year. Similar cases have been identified in developing countries including China, with increasing costs associated with the aged population^2^. Percutaneous Vertebroplasty (PVP) and Percutaneous Kyphoplasty (PKP) are minimally invasive treatments that apply bone cement to strengthen a fractured vertebral body. Their main advantages include a rapid pain relief, correcting kyphosis deformity, improvement of the physical function and daily activities^3–8^. Therefore, many surgeons consider them as effective strategies for treating osteoporotic fractures^9^. With the increase of interventions, surgeons have detected higher risks of additional fractures in adjacent non-treated levels, following augmentation with bone cement after PKP and PVP^10,11^. Among the risk factors associated with new fractures in adjacent vertebra occurring in patients underwent PVP, several investigators have identified low bone mineral density, the amount of cement injected, the restoration of vertebral height, vitamin D deficiency, and the intradiscal cement leakage (ICL)^12–17^. It has been speculated that intradiscal cement leakage (ICL) represents one of the independent risk factors of adjacent vertebrae fracture after PKP^12,13,18,19^.

Up to the present, no research has confirmed how ICL affects the biomechanics of adjacent segments. The present study establishes finite element models of thoracolumbar OVCF before and after PKP with intraoperative cement leakage in the disc from CT images. Our aims were: (a) to investigate the biomechanical effects on adjacent vertebra, and (b) to evaluate the risk of subsequent fractures in adjacent vertebral bodies with respect to leakage of cement in the disc during PKP.

## Materials and methods

### Patient

**Figure 1:**
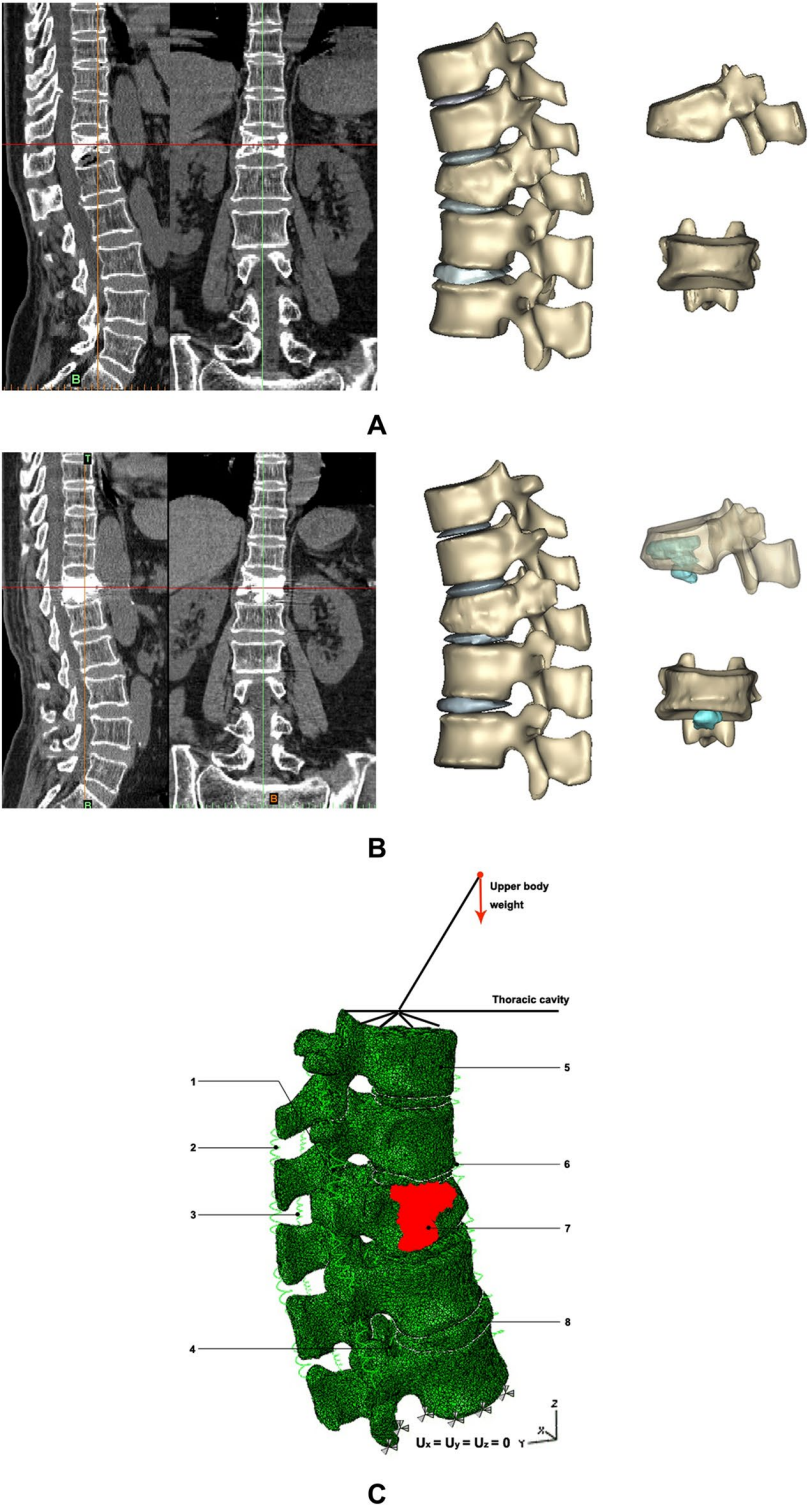
(**A**) CT imaging (sagittal and coronal view) and three-dimensional (3D) model of the T10–L2 section prior to PKP. (**B**) CT imaging (sagittal and coronal view) and 3D model of the T10–L2 section after PKP operation and the leakage cement (blue color). (**C**) Finite element model of the T10–L2 section: (1) intertransverse ligament; (2) supraspinal ligament; (3) interspinal ligaments; (4) posterior longitudinal ligament; (5) vertebral body(VB); (6) anterior longitudinal ligament; (7) bone cement; (8) intervertebral disc.

A 71-year-old female patient who had suffered from persistent low back pain for two months after an accidental fall, which could not be relieved by expectant treatment, was investigated. X ray, Computed tomography (CT) and Magnetic Resonance Imaging (Acute single segmental OVCF (Magnetic resonance imaging [MRI] on T1-weighted images showed low signal. MRI on T2-weighted images and short tau inversion recovery sequences showed high signal) confirmed a single T12 vertebra compression fracture. BMD of left femoral neck measured by dual-energy X-ray absorptiometry was − 2.9. PKP was conducted by means of unilateral biopsy needles in the vertebral body adopting a transpedicular approach. The needles were subsequently replaced by working cannulas. Working channels were prepared to allow balloons to be introduced and stepwise inflated with controlled volume and pressure. After the removal of the balloons, 4 ml viscous Polymethylmethacrylate (PMMA) was injected in the cavity. The procedure was performed under local anesthesia and fluoroscopy with C-arms. Cement leaking into the disc between T12 and L1 vertebrae was found soon after the real time fluoroscopy in the AP and lateral projections were checked. No pain was reported after the intervention. X-ray and CT images before and after operation were collected with the patient’s signed informed consent (as shown in Fig. 1).

### Finite element modeling

The finite element modeling consists of five main steps: geometrical, material, element type, load modeling, and validating the complete model^20,21^.

All data were obtained from CT scans (485slices) of T10–L2 vertebra with transverse sections of 1.0 mm thickness, 1.0 mm space, tube voltage 120 kV, tube current 200 mA. CT images were transferred from the database in DICOM format, and then analyzed with MIMICS 10.01 software (Materialise’s Interactive Medical Image Control System, Materialise, Inc., Leuven, Belgium) , to build a three-dimensional model (as shown in Fig. 1). Vertebrae and cement were extracted by HU value^22^. (It is mathematically defined as, ρ = 1.122 * (HU) + 47 (1), E = 0.02 * ρ1.69 (2)).

The cortical shell, cancellous core, posterior bony elements and the bony endplates of the vertebral bodies are generally distinguished. Due to the practical difficulty in distinguishing the intervertebral discs and vertebras from CT scan of osteoporosis patients, we manually drew out the discs slice by slice, calculated the 3D geometrical models, and then measured and divided the disc nucleus and annulus in the ABAQUS 6.9 software (Dassault Systèmes Simulia Corp., Providence, RI, USA)^23^. Intervertebral discs were built according to anatomical structure, which were represented as degenerated state and simulated as an inner disc nucleus surrounded by disc annulus^24^. The volumetric relation between annulus and nucleus is nearly 3:7. Seven different ligaments (i.e. interspinous, supraspinous, intertransverse, posterior longitudinal, capsular, anterior longitudinal and ligamentum flavum) were distinguished.

Three finite element models were established.

#### Model A

CT data were collected before the PKP procedure. The three-dimensional finite element models consisted of T10 vertebra (70,747 elements), T11 vertebra (75,203 elements), T12 vertebra (73,708 elements), L1 vertebra (94,067 elements), L2 vertebra (102,302 elements), T10–T11 intervertebral disc (94,067 elements), T12–L1 intervertebral disc (33,573 elements), and L1–L2 intervertebral disc (23,502 elements). The model consisted of 492,970 elements, and the element type is C3D4.

#### Model B

CT data were collected after the PKP procedure. The three-dimensional finite element models consisted of T10 vertebra (67,388 elements), T11 vertebra (72,172 elements), T12 vertebra (84,013 elements), L1 vertebra (107,274 elements), L2 vertebra (99,612 elements), T10–T11 intervertebral disc (107,274 elements), T12–L1 intervertebral disc (33,079 elements), and L1–L2 intervertebral disc (24,812 elements). The intact model consisted of 509,803 elements, and the unit type is C3D4.

#### Model C

Same as model B except the T12–L1 intervertebral disc without intradiscal cement leakage (ICL).

The material properties of the finite element models’ components, such as vertebrae (endplate, cancellous, and cortical), degenerated disc, and bone cement, were extracted from previous studies^25,26^, and reported in Table 1. It was assumed that all the components were isotropic and homogenous. All seven major spinal ligaments of the lumbar spine were included in the model, represented by spring elements with a linear stress strain-behavior and the restraint capacity to transmit tensile forces^27,28^ (as shown in Table 2). The interaction between the intervertebral disc and the endplates were set as tied interface (above and below). The interaction property of “finite sliding” feature was used to define the interactions between two articular processes, which allowed for small relative displacements between two deformable contacting surfaces. The coefficient of friction is 0.2. Based on published studies, a 400-N compressive load was found to be representative of compressive loading in vivo while standing^29,30^. The load was applied as a uniform compressive pressure of 0.3 MPa on the superior endplate of T10. The standard load cases flexion–extension, axial rotation and lateral bending of the upper body were simulated by 15 Nm moment load on the finite element models^31–33^. Boundary conditions included constraining the base of the lower vertebral body (L2) in all degrees of freedom (U1 = U2 = U3 = UR1 = UR2 = UR3 = 0). The von Mises stress data during different loading conditions were collected (as shown in Fig. 1).

**Table 1:** Element type and values of properties of the tissues and material in the finite element model.

Tissue/material	State	Element type	Thickness (mm)	Modulus of elasticity (MPa)	Poisson’s ratio
Vertebra					
Cortical bone		Shell	0.4	5000	0.2
Cancellous bone	Normal	C3D4*	N/A*	100	0.2
	Osteoporosis	C3D4	N/A	25	0.2
Endplate		Shell	0.25	1000	0.2
Posterior bone			N/A	3500	0.25
Intervertebral disc					
Annulus fibrosus	degenerated	C3D4	N/A	8.4	0.45
Nucleus pulposus	degenerated	C3D4	N/A	8.4	0.45
PMMA bone cement		C3D4	N/A	4000	0.33

*C3D4 = 4-noded hex, *N/A* not applicable.

**Table 2:** Properties of the ligaments included in the finite element model.

Major ligaments	Modulus of elasticity (MPa)	Cross section area (mm^2^)	Mean length (mm)	Rigidity* (N/mm)
Anterior longitudinal	7.8	22.4	20	8.74
Posterior longitudinal	10	7.0	12	5.83
Ligamentum flavum	17	14.1	15	15.38
Transverse	10	0.6	32	0.19
Capsular	7.5	10.5	5	15.75
Interspinous	10	14.1	13	10.85
Supraspinous	8.0	10.5	22	2.39

*Rigidity = Elastic modulus × Cross-Section Area/length.

The ABAQUS 6.9 software (Dassault Systèmes Simulia Corp., Providence, RI, USA.) was used to construct and analyze the model. The output variables included the maximum and minimum principal stresses in the endplate and cancellous and the cortical bone of each vertebra. In addition, we examined overall and axial model deformation and compared the data of the models treated with those untreated. The three finite element models were validated by comparing the correlation curve of axial compressive load with the experimental results in the same boundary conditions in vitro data from the literatures^18,26,28,30,31^.

## Results

### The effects of von Mises stress on adjacent vertebrae before and after PKP with/without intradiscal cement leakage (ICL)

The von Mises stress constitutes a major part for mechanics analysis, including the maximum principal stress, and the distribution of Von Mises stress on adjacent endplate and intervertebral discs before and after PKP.

**Figure 2:**
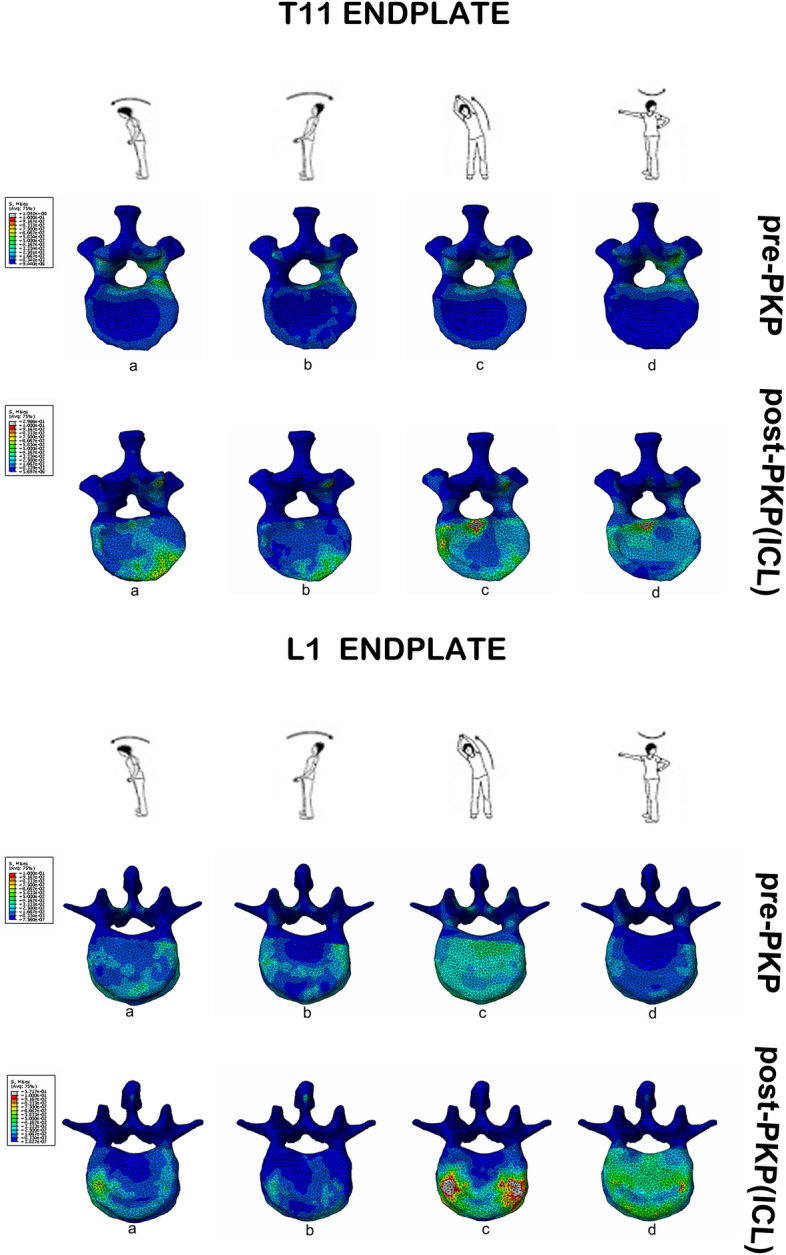
Von Mises stress contours on the adjacent endplates (that is, endplates at T11 and L1) prior to and after BKP: a. anteflexion, b. backward extension, c. left lateral bending, d. counterclockwise-acting axial rotation.

The distribution of von Mises stress of the adjacent endplate remained asymmetrical before and after the operation. The maximum principal stress increased and the Von Mises stresses distribution of adjacent endplates changed after PKP. The von Mises stress hotspot was enhanced especially on lateral bending and twist during loading conditions due to the intradiscal cement leakage (ICL) (as shown in Fig. 2).

### The maximum von Mises stresses of vertebrae before and after PKP with/without intradiscal cement leakage (ICL)

**Figure 3:**
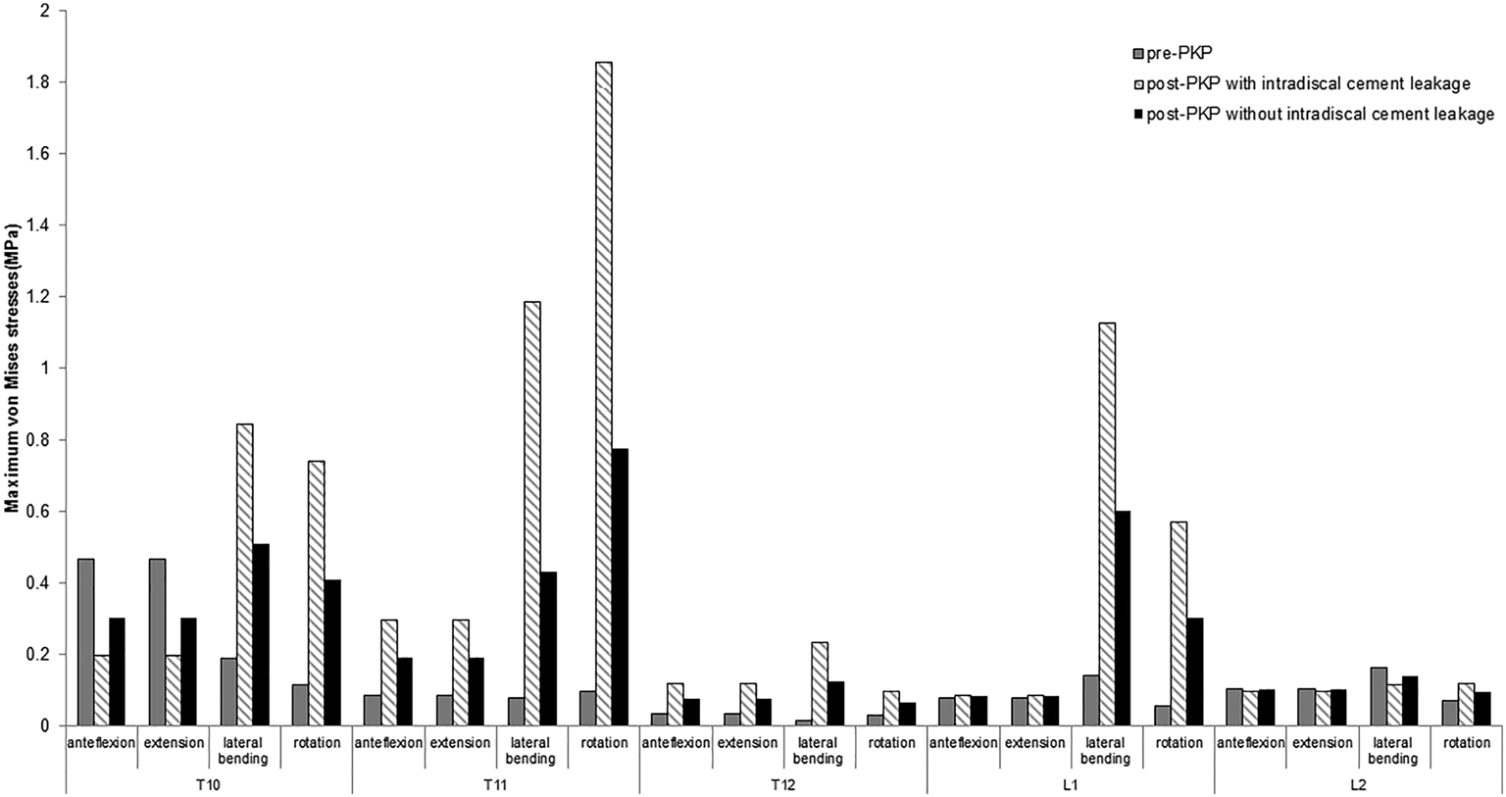
A summary of the maximum von Mises stresses in the augmented VB (T12) and in the unaugmented adjacent VBs (T10, T11, L1, and L2).

Leakage of cement in the disk during PKP increases the maximum von Mises stress of adjacent vertebral bodies including endplates under flexion, extension, lateral bending and rotation loading conditions (as shown in Fig. 3). Especially for lateral bending and rotation loading conditions, the maximum von Mises stress increased of 1455.94% and 1831.79% for T11 vertebra, 702.06% and 931.56% for L1 vertebra compared with those before the intervention. However, for the distal non-treated vertebrae such as T10 and L2, the maximum von Mises stress with anteflexion and backward extension loading conditions decreased of 57.73% and 57.64% for T10 vertebra and of 6.75% and 6.71% for L2 vertebra. By contrast, the maximum von Mises stress of adjacent vertebral bodies of PKP without intradiscal cement leakage (ICL) does not increase so obviously.

### Validation result for functional spinal unit (FSU) L1–2 of model A, B, C

**Figure 4:**
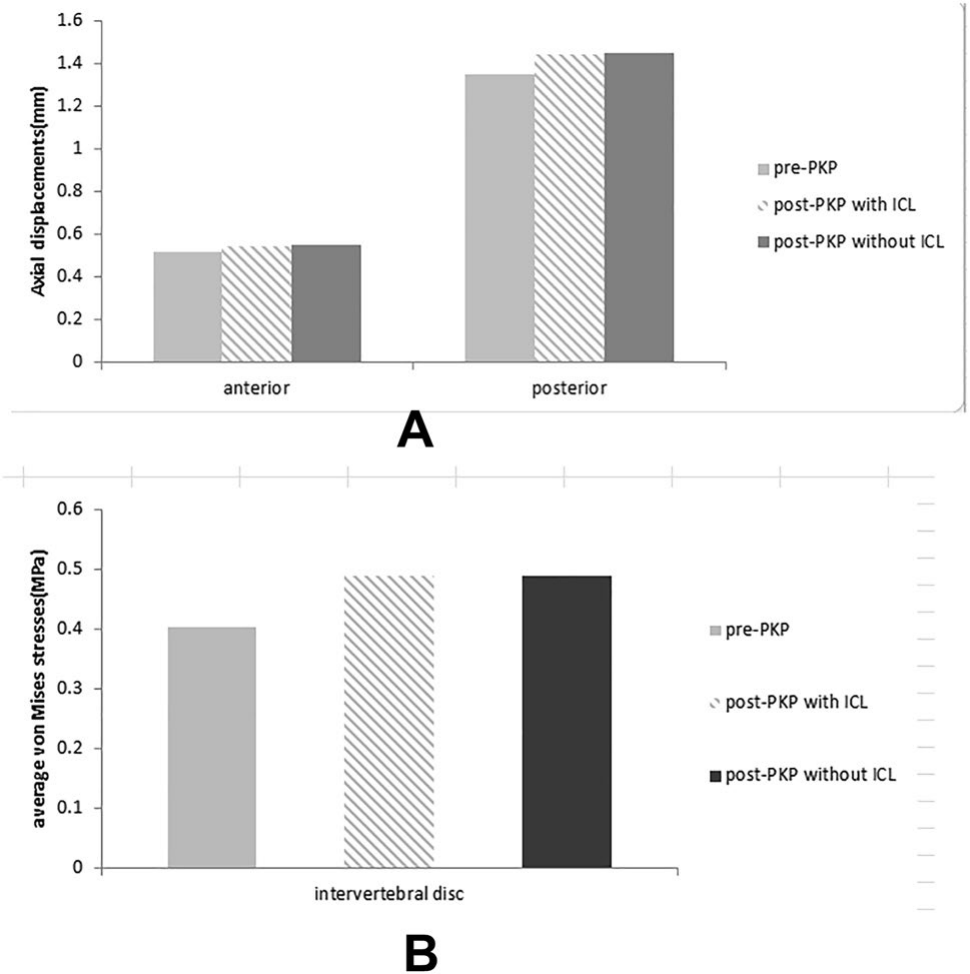
(**A**) Axial displacements of anterior, posterior columns of A, B, C models (pre-PKP, post-PKP with ICL, post-PKP without ICL) under an axial compressive load of 400 N; (**B**) average von Mises stresses in the intervertebral discs A, B, C models (pre-PKP, post-PKP with ICL, post-PKP without ICL) under an axial compressive load of 400 N.

Due to the lack of previous biomechanical researches on five vertebral of osteoporotic fracture model in vitro, we chose functional spinal unit (FSU) L1–2 for the validation. With an axial compressive load of 400 N, the axial displacement values of the anterior and posterior columns of the three models are shown in Fig. 4A. The range of axial displacement 0.52–1.45 mm fell within the range of experimental results (0.26–1.5 mm) reported before^30,34,35^.

Meanwhile, the average disc von Mises stresses of the three models were 0.403 MPa, 0.490 MPa and 0.490 MPa respectively, deviating little from the previous result of 0.48 MPa^30,36^ (as shown in Fig. 4B).

## Discussion

Percutaneous kyphoplasty is accepted as a safe and efficient therapeutic strategy for patients suffering from untreatable pain and disability caused by an osteoporotic vertebral fracture. The vertebroplasty provides immediate pain relief and stabilization, and is characterized by a high rate of successful treatments with low morbidity, hospitalization and adverse events. However, while vertebroplasty greatly increases the failure strength of augmented vertebrae, a significantly increased risk of adjacent vertebral body fractures was recognized in both clinical and experimental studies^10,16,19,37^. Besides, patients were reported to suffer from recurrent pain. The mechanism for adjacent vertebral fractures remains unclear, and it is still uncertain whether adjacent vertebral body fractures are related to the natural progression of osteoporosis or caused by complications of the procedure of kyphoplasty^26,36^. Therefore, our studies provided a better understanding of the immediate biomechanical effects of adjacent vertebral bodies after kyphoplasty, and compared this biomechanical state with that already present due to the natural progression of osteoporosis.

Rho et al*.* and Lin et al*.* reported that leakage of cement into the intervertebral disc during augmenting vertebrae with bone cement increases the risk of new fracture of adjacent vertebral bodies^12,18^, although biomechanical evaluations have not been conducted. In vitro experimental measurements on spinal components are mainly limited by the poor availability of samples and the variability among the specimens. In vivo measurements provide information on the spine in its natural state, but are restricted by the requirement for limited invasiveness. More recently, a third approach called “in silico” testing has become popular in assessing the biomechanical state of the spine. Finite element analysis has been developed as an improved method for investigating the biomechanical effects on adjacent vertebra after PKP with intradiscal cement leakage (ICL)^28^.

Several years ago, we investigated a case of cement leakage in the disc after PKP which occurred during our clinical research. By using pre- and post-operative CT images, we developed finite element models of spines composed of four functional spinal units (FSU), and set axial loading to evaluate the influence of intradiscal cement leakage (ICL) of kyphoplasty on the biomechanics of treated vertebral bodies and adjacent non-treated vertebral bodies^38^. During the process, we found something more interesting, so in this research, we highly improve the models, so that we can simulate all kinds of physiological activities (flexion, extension, lateral bending and rotation) to approach more real life situations. Then we found the intradiscal cement leakage and the distribution of the bone cement could change the biomechanical effect of adjacent vertebrae, and lead to extremely high von Mises stresses of adjacent vertebra in some kinds of physiological activities (such as lateral bending and rotation in this research), which may increase the risk of new fracture of adjacent vertebral bodies.

The data of the two models were obtained from computer tomography (CT) scans of T10–L2 vertebra before and after PKP, based on which the three-dimensional model was built separately. So there may be some differences between the vertebrae and intervertebral discs of the two models. In fact, previous studies found twenty-one percent of vertebrae had new areas of marrow edema on follow-up. Twenty-two percent of vertebrae imaged 6 months after vertebroplasty had moderate or severe edema, progressive and persistent edema and interval height loss after successful vertebroplasty are common^39^. The models were designed to simulate physiological movements such as flexion, extension, lateral bending, and twisting with different loads, in order to explore the stresses of adjacent and distal non-treated vertebrae. Comparative studies of the data before and after PKP with/without intradiscal cement leakage (ICL) led us to examine the causes of adjacent vertebral body fractures and whether the fractures were resulted from natural progression of osteoporosis or intradiscal cement leakage (ICL).

We found that the maximum von Mises stresses on adjacent vertebrae increased with different positions compared with those before PKP, especially on lateral bending and twisting loading conditions. In addition, the maximum von Mises stresses of the distal non-treated vertebrae changed under lateral bending and twisting loading conditions, and even decreased on anteflexion and backward extension loading conditions. Furthermore, the Von Mises stresses distribution of adjacent endplates also changed after PKP, and the Mises stress hotspot was enhanced. We have checked the mesh quality in these regions before load was applied, the mesh quality of which is the same as other’s. In fact, compared with the model of kyphoplasty without intradiscal cement leakage, the regions of the maximum von Mises stress were different. The data we collected is from a real case, the cement leakage in the disc is very irregular, and the regions with high von Mises stress in lateral bending simulation after PKP may be just against the protruding part of it, which may have caused such an extreme high Von Mises stress in adjacent vertebra and led adjacent vertebra compression fractures. Due to the wage differences in the material properties between bone cement and Intervertebral disc, the chance of adjacent Vertebrae fatigue failure may highly increase after frequent movements, which we will test and verify in later researches. The process of cumulative “fatigue failure” of the vertebral body has been investigated in human cadaver spines^40–42^.

Compared with the change of PKP model without leakage of cement, our research shows that the cement leaking into the disc changes the stress distribution of the vertebrae, substantially increases the von Mises stresses of adjacent vertebra, which hints the cause of adjacent vertebra compression fractures. Previous clinical studies have found the increase incidence of new adjacent vertebra fractures^13,16,26^, and in some researches, bone cement leakage, especially intradiscal cement leakage(ICL) presented highly correlated with the new fractures of adjacent vertebrae^43–47^, however the exact relationship between fractures and ICL is still not very clear. In our researches, we have shown the biomechanical changes of adjacent vertebrae after PKP, that intradiscal cement leakage can increase the maximum von Mises stress on adjacent vertebrae, change the distribution of von Mises stress on adjacent endplates, and enhance their hotspot. Those indicates that the biomechanical changes after ICL may play an important role on failure of adjacent vertebrae after PKP. The different results and regions of the maximum von Mises stress in PKP with and without intradiscal cement leakage may suggest the important role of the shape, size and distribution of the bone cement, which will be our next-stage researches.

However, our approach has some limitations, as it is based on a single clinical case. This requires further comparative studies to confirm the analysis and prediction, and the effect of those increases needs to be confirmed by additional mechanical testing in the future. Moreover, there are many ways to build the three-dimensional finite element models, and we do not claim ours to be the best. The material properties of the models came from published literature instead of in vivo data, which may have undermined the accuracy of results.

Hiwatashi et al. found cement leakage into an adjacent disk is more common when there are cortical defect in the endplate of the treated vertebral body, abnormal T2 hyperintensity in the adjacent intervertebral disk, and absence of intravertebral cleft^48^. So that preoperative MRI is a good choice for predicting such cement leakage. The reduction of the incidence of cement leakage in the disc during kyphoplasty is also very important, and cement injection should be stopped once leakage in the disk is detected. For patients with intradiscal cement leakage (ICL), careful and frequent follow-up observation is necessary. Whether the upper and lower adjacent vertebra should be augmented as a prophylactic treatment to reduce the risk of postoperative fracture still needs further investigation.

### Limitations

1. Only one results of stress is included in our research, it still needs further study.
2. Whether the upper and lower adjacent vertebra should be augmented as a prophylactic treatment to reduce the risk of postoperative fracture still needs further investigation.

## Conclusion

Post-operative adjacent vertebra compression fractures after PKP for Osteoporotic Vertebra Compression Fracture (OVCF) with intraoperative intradiscal cement leakage (ICL) may be closely related to the biomechanical changes of adjacent vertebrae of thoracolumbar OVCF, and it may increase the risk of postoperative fracture.

## Data Availability

The data used to support the findings of this study are included within the article.
